# TPGS-1000 exhibits potent anticancer activity for hepatocellular carcinoma *in vitro* and *in vivo*

**DOI:** 10.18632/aging.102704

**Published:** 2020-01-27

**Authors:** Yidan Chen, Liqin Mo, Xuan Wang, Bi Chen, Yunfen Hua, Linyan Gong, Fei Yang, Yongqiang Li, Fangfang Chen, Guiting Zhu, Wei Ni, Cheng Zhang, Yuming Cheng, Yan Luo, Junping Shi, Mengsheng Qiu, Shixiu Wu, Zhou Tan, Kaifeng Wang

**Affiliations:** 1 Cancer Research Institute, Hangzhou Cancer Hospital, Zhejiang, China; 2 Life Sciences Research Institute, College of Life and Environmental Sciences, Hangzhou Normal University, Zhejiang, China; 3 Oncology Department, The Affiliated Hospital of Hangzhou Normal University, Zhejiang, China; 4 College of Pharmaceutical Science, Zhejiang University of Technology, Zhejiang, China; 5 State Key Laboratory for Oncogenes and Related Genes, Department of Oncology, Renji Hospital, School of Medicine, Shanghai Jiaotong University, Shanghai Cancer Institute, Shanghai, China

**Keywords:** hepatocellular carcinoma, TPGS1000, apoptosis, migration, malignancy

## Abstract

D-alpha-tocopheryl polyethylene glycol 1000 succinate (TPGS1000) is the most active water-soluble derivative of vitamin E and has been widely used as a carrier of solvents, plasticizers, emulsifiers, absorbent agents and refractory drug delivery systems. However, its anti-hepatocellular carcinoma (HCC) properties have not been explored. HCC cells were treated with different concentrations of TPGS1000. Cell survival was tested by CCK8 assay, and cell migration was tested by wound healing and Transwell assay. EdU staining verified cell proliferation, and signalling pathway was assayed by Western blot analysis. The BALB/c-nu mouse xenograft model was established to test HCC cell growth *in vivo*. *In vitro* TPGS1000 significantly inhibited the viability and mobility of HCC cells (HepG2, Hep3B and Huh7) in a dose-dependent manner. Cell cycle analysis indicated that TPGS1000 treatment arrested the HCC cell cycle in the G0/G1 phase, and induction of cell apoptosis was confirmed by TUNEL and Annexin V-7-AAD staining. Further pharmacological analysis indicated that collapse of the transmembrane potential of mitochondria, increased ROS generation, PARP-induced cell apoptosis and FoxM1-p21-mediated cell cycle arresting, were involved in the anti-HCC activity of TPGS1000. Moreover, treatment *in vivo* with TPGS1000 effectively impaired the growth of HCC xenografts in nude mice.

## INTRODUCTION

Hepatocellular carcinoma (HCC) is the sixth most common cancer worldwide and the fourth leading cause of cancer mortality [1]. Despite improvements in diagnosis and clinical treatment strategies, the 5-year survival rate for HCC is less than 17% for all stages combined [2]. Currently, the most important treatment for HCC is chemotherapy, and Sorafenib remains the most conventionally used first-line drug for treatment of HCC [3, 4]. However, the development of multidrug resistance (MDR) in HCC can dramatically reduce the efficacy of chemotherapy, which results in no consistently effective treatment for HCC [5]. Other anti-HCC drugs, such as camptothecin, are not water-soluble and are not easily absorbed by the human body, which limits their therapeutic effect [6]. Hence, there is an urgent need for more effective therapies or synergistic agents for the treatment of liver cancer.

**Figure 1:**
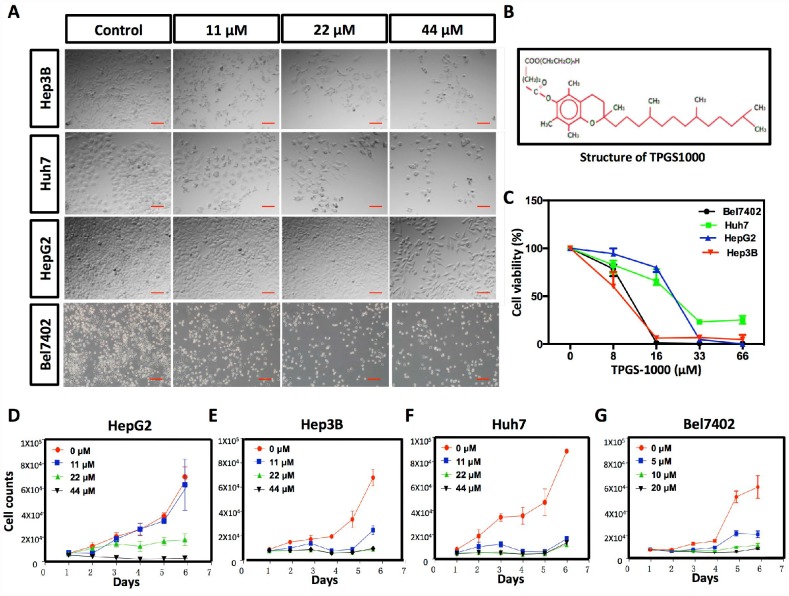
**Effect of TPGS1000 on viability and proliferation of hepatocarcinoma cells.** (**A**) Morphology of four HCC cell lines (HepG2, Hep3B Huh7 and Bel7402) treated with different concentrations of TPGS (0, 11, 22 and 44 μM), scale bar = 100 μm. (**B**) The structure of TPGS1000. (**C**) HCC cells were cultured in the presence of various concentrations (0–66 μM) of TPGS for 48 h. Cell viabilities were measured using CCK8. The line graph represents the percent viable cells compared to the vehicle-treated cells. (**D**–**G**) Growth curves of four HCC cell lines were plotted from cell counts under different concentrations of TPGS.

D-alpha-tocopheryl polyethylene glycol 1000 succinate (TPGS1000) is a water-soluble derivative of natural vitamin E [7, 8]. Its amphiphilic structure consists of a lipophilic alkyl tail and a hydrophilic polar head (Figure 1A). Since it was first approved as a safe pharmaceutical adjuvant by the US FDA and the European Medicine Agency (EMA) for human use [9], TPGS has been extensively researched and used as a solubilizer, a pore-forming agent, and a bioavailability enhancer for hydrophobic drugs in various drug delivery systems [10]. Furthermore, TPGS has been reported to synergistically enhance the cytotoxicity of many anticancer drugs [11, 12] and inhibit the function of P-glycoprotein (P-gp), a protein that causes MDR by acting as a drug efflux pump [13]. TPGS alone also showed inducing apoptosis in lung cancer [14]. While its anticancer efficacy in liver cancer have not been explored.

The current study was aimed at evaluating TPGS *in vitro* for its cytotoxic properties against human liver cancer cell lines (HepG2, Hep3B, Huh7 and Bel7402), and also *in vivo* for its inhibition of xenograft tumor progression by either direct delivery or by administration through the digestive or circulatory system. Accompanied with interpretations of the possible underlying mechanisms, our findings suggest that TPGS could not only be used as a P-gp inhibitor to reverse MDR but also to enhance its potential therapeutic efficacy against HCC via its unique mechanisms.

## RESULTS

### TPGS1000 suppressed the viability and proliferation of HCC cells

The effects of TPGS treatments (0, 11, 22 and 44 μM) on HCC cell viability were examined in the HCC cell lines HepG2, Hep3B Huh7 and Bel7402. TPGS treatments lead to significant decreases in the number of cells and to a remarkable change in the shape of the HCC cells as well. Untreated cells appeared to have large cell bodies with a polyhedral shape. TPGS-treated cells were relatively thinner and contained many intracellular vacuoles (Figure 1A). To quantify the effect of TPGS on the viability of HCC cells, CCK8 assays were performed. We observed that TPGS treatments (0-66 μM) dose-dependently reduced the viability of HCC cells (Figure 1C). The IC50 values for TPGS were 22.34 μM, 8.67 μM, 10.7 μM and 17.08 μM in HepG2, Hep3B, Bel7402 and Huh7 cells, respectively. In parallel, cell growth curves were plotted from cell counting data and demonstrated the inhibition of HCC cell growth over time by TPGS treatments (Figure 1D–1G). It is apparent that 11 μM TPGS was sufficient for arresting Hep3B and Huh7 cell proliferation (Figure 1E and 1F) and that Bel7402 are more sensitive to TPGS than HepG2 (Figure 1G and 1D).

### TPGS restrained the migration and invasion of HCC cells

**Figure 2:**
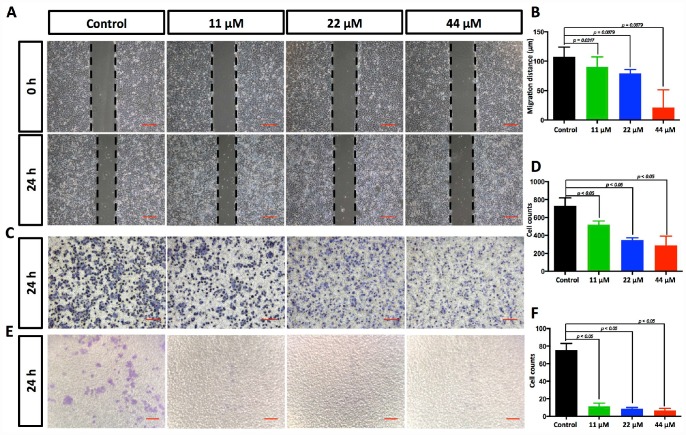
**TPGS dose dependently restrained HCC cell migration and invasion.** (**A**) Effects of TPGS treatments on HCC cell migration, scale bar = 100 μm (**B**) The migration distance of HCC cells was quantified by ImageJ software, and the 44 μM TPGS group had the shortest migration distance (23 μm). (**C**) The inhibition of HCC cell migration by TPGS was confirmed by Transwell assays, scale bar = 100 μm. (**D**) The migrated cells were counted after Crystal violet staining with the 44 μM TPGS group having the lowest number of migrated cells (approximately 298). (**E**) TPGS diminished cell invasion of HCC cells (Transwell assay using an 8 μm pore filter coated with 0.5 mg/mL Matrigel), scale bar = 100 μm. (**F**) The mean cell counts of invading cells, with the 44 μM TPGS group having the lowest number of invasion cells (approximately 6).

To determine the functional impact of TPGS treatments on HCC cells, we next examined the effects of TPGS on the 2D- and 3D-migration and the 3D-invasion of HCC cells by wound-healing (Figure 2A and Supplementary Figure 1A, 1B) and Transwell assays (Figure 2C and 2E and Supplementary Figure 1C–1F). Wound healing involves a number of processes, including cell proliferation, migration and the establishment of cell polarity [15]. To limit the impact of cell growth on our wound-healing assay, we starved the cells before and during the wounding assay of the monolayer cells. As shown in Figure 2B, the 2D-migration distances were reduced in a dose-dependent manner after TPGS treatments (*p* < 0.05), and the 44 μM group had the shortest migration distance (approximately 23 μm). Furthermore, this 2D-migration restraint of HCC cells was confirmed by 3D-migration assays using uncoated Transwells (Figure 2C). As shown in Figure 2D, the number of HCC cells that passed through the filter decreased significantly as the TPGS concentrations increased (*p* < 0.005). Since cell invasion is important for HCC metastasis [16], the reduction in invasive cell numbers (from approximately 75 to 6) through the Matrigel-coated Transwell membranes indicated that TPGS treatment attenuated not only the viability but also the motility of the HCC cells (Figure 2E and 2F).

### TPGS inhibits HCC cell proliferation by arresting the cell cycle in the G0/1 phase and promotes cells into late apoptosis

**Figure 3:**
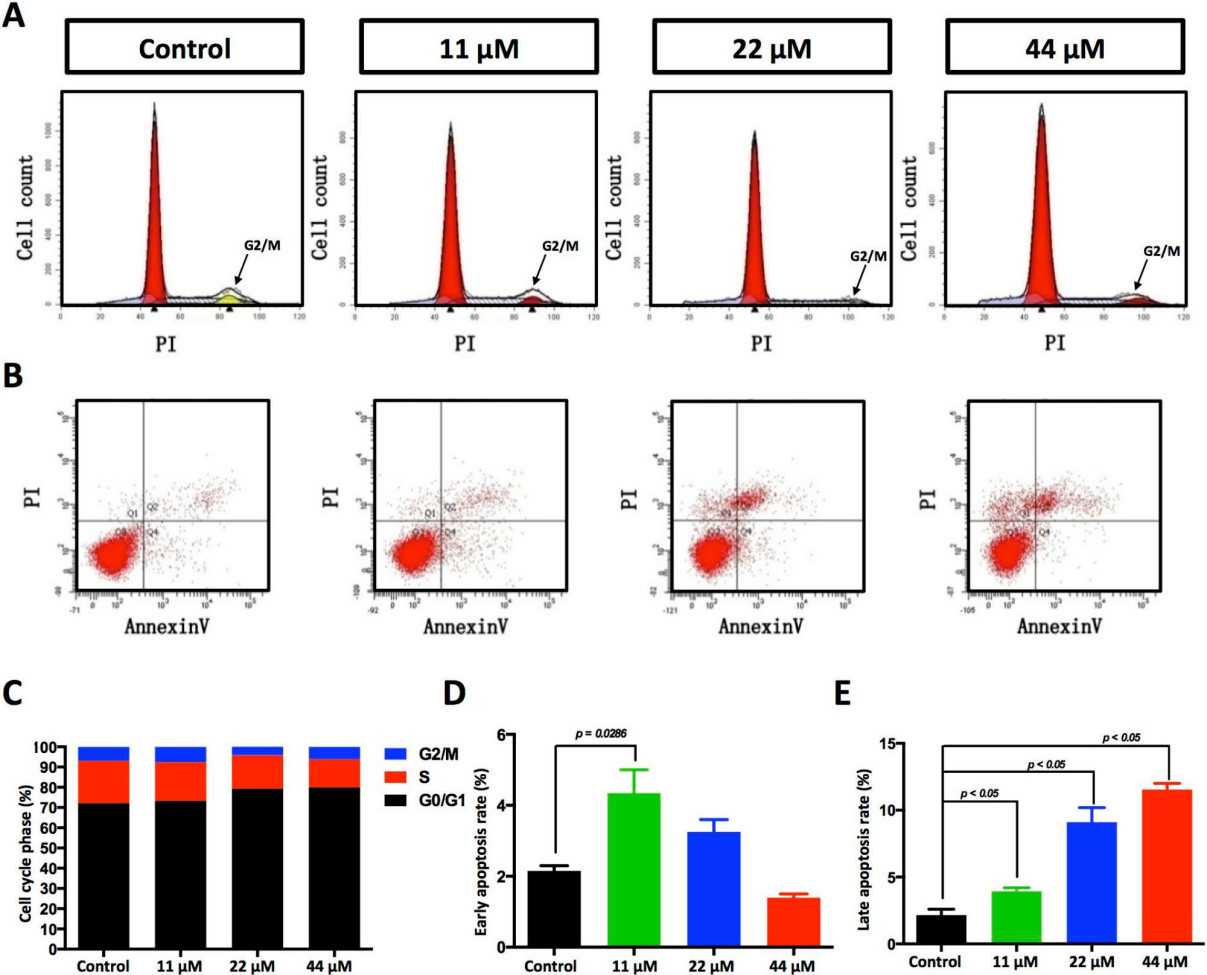
**Effects of TPGS treatments on HCC cell cycle progression and cell apoptosis.** (**A**) Cell cycle progression detected by FACS analysis. Cells in G0/G1 are marked in the red area. Cells in the S phase are marked with a slash, whereas the arrowhead indicates the G2/M cells. (**B**) Cell apoptosis was assessed with Annexin V-PI staining. (**C**) The cell cycle distribution was calculated with Cell Quest Pro software. The 44 μM TPGS group produced the lowest amount of cell accumulation in the S phase (13.88%). (**D**) The HCC cells that were treated with 11 μM TPGS had the highest ratio of early apoptotic cells (approximately 4%), whereas with increasing TPGS concentrations in the HCC cells, the 44 μM TPGS group had the highest late apoptotic ratio, 10.4% (**E**).

**Figure 4:**
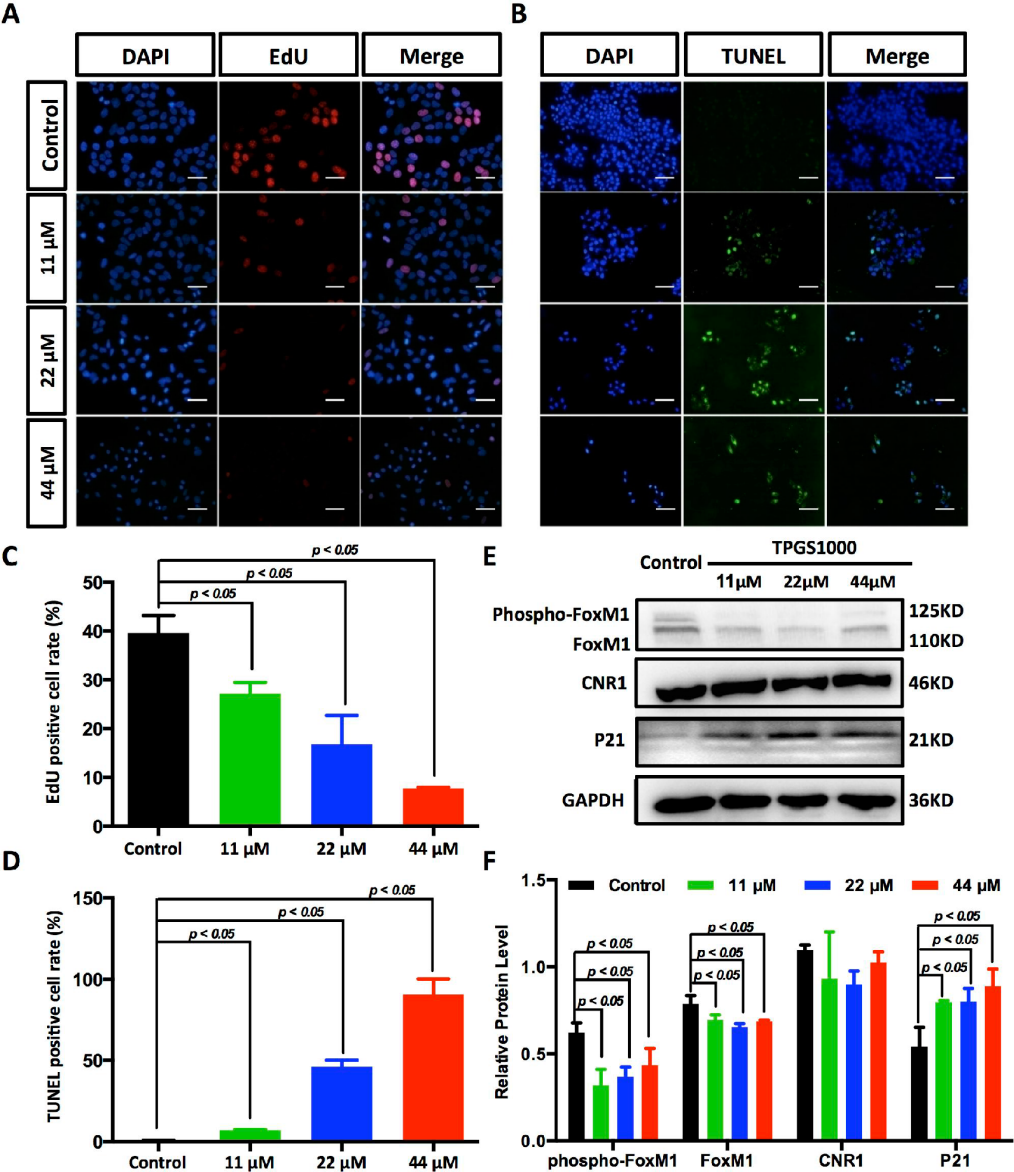
**Suppression of DNA synthesis and induction of apoptosis in TPGS-treated HCC cells.** (**A**) Detection by fluorescence microscopy of EdU (red) incorporated into the DNA of cultured HCC cells, scale bar = 40 μm. The nuclei were counter-stained with DAPI (blue). (**B**) TUNEL (green) positive apoptotic cells in HCC cells induced by TPGS treatments, scale bar = 20 μm. (**C**) The rates of EdU positive cells that passed through the S phase were calculated with ImageJ, and the 44 μM TPGS group had the lowest EdU positive cell rate (7%). (**D**) The rates of TUNEL positive cells were elevated with increasing TPGS concentrations, and the 44 μM TPGS group had the highest apoptotic cell rate (approximately 93%). (**E**) A decrease of FoxM1 and phosphorylated FoxM1, and an increase of p21 protein levels in TPGS-treated HCC cells. (**F**) Quantitative analysis of western blot results from (**E**). All protein levels were normalized with the housekeeping genes GAPDH and β-actin.

In our experiments, HCC cell proliferation was dose-dependently suppressed by TPGS (Figure 1D–1G). To uncover the underlying mechanisms, we investigated the effect of TPGS on HCC cell cycle control by cell cycle profiling (Figure 3A). Treatment with 22 or 44 μM TPGS resulted in an increase of HepG2 cells in the G0/1 phase compared with the untreated or 11 μM groups. The G0/1 cell cycle arrest then led to a decrease of cells in the S phase (Figure 3C). Furthermore, the induction of apoptosis was examined by enhanced Annexin V-PI staining. The TPGS-treated HepG2 cells exhibited enhanced Annexin V staining (Figure 3B) as well as irregular nuclear morphologies (Figure 4A, 4B), which were further quantified by Cell Quest Pro software. Treatment of HepG2 cells with TPGS for 24 h resulted in a dose-dependent increase in late apoptosis from 2.5% to 10.4% (Figure 3E). In addition, the early apoptotic rate of 11 μM TPGS-treated cells increased at first, and then decreased in late apoptosis expansions (Figure 3D).

### TPGS inhibits HCC cell proliferation by repressing DNA synthesis and effectively inhibit FoxM1 signalling pathways

The decreased cells in S phase and the increased cellular apoptosis were confirmed by EdU uptake assay and TUNEL staining (Figure 4A, 4B and Supplementary Figure 2A, 2B). DNA replication occurs in the S phase of the cell cycle and is crucial for the proliferation of cancer cells. Using EdU labelling (red) as an S phase marker of proliferating cells (Figure 4A), we found that the percentage of DNA-synthesizing cells (EdU positive cells) decreased from 38.3% to 7.0% (Figure 4C). With TUNEL assays, as shown in Figure 4B and 4D, the percentage of apoptotic HCC cells that became labelled with green fluorescence increased with the dose of TPGS (the rate of TUNEL positive cells increased from 0.5% to 92.6%).

To determine potential drug targets of TPGS, we utilized the Swiss Target Prediction [17], and the predicted targets that were obtained are listed in Supplementary Table 2. We determined that the expression of the most likely drug target, Cannabinoid receptor 1 (CNR1), did not respond to TPGS treatments (Figure 4E). However, for one of its potential downstream molecules, Forkhead Box M1 (FoxM1) [18], the levels of both phosphorylated FoxM1 (phosphor-FoxM1) and FoxM1 itself decreased after treatment with TPGS (Figure 4F). Several studies have shown that targeting FoxM1 is an effective therapeutic approach against liver cancer, and FoxM1 downregulation or inactivation leads to inhibition of proliferation, migration and invasion of various cancer cells [19, 20]. Furthermore, we also observed decreased expression and phosphorylation of FoxM1 with a concomitant increase (greater than 50%) in p21 proteins (Figure 4E, 4F and Supplementary Figure 3A, 3B), suggesting an important role for FoxM1-p21 signalling in HCC cell cycle progression and tumorigenesis.

### TPGS increased the production of ROS and attenuated the cellular mitochondrial membrane potential

**Figure 5:**
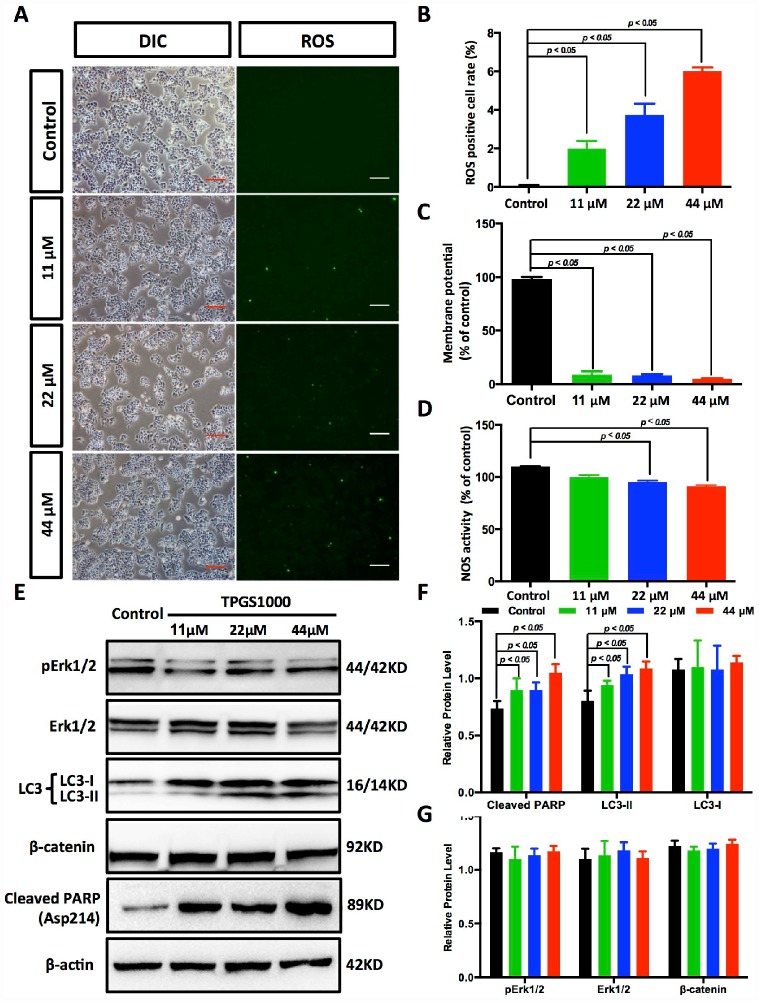
**TPGS dose dependently induced the production of ROS and reduced energy production in HCC cells.** (**A**) ROS imaging (green fluorescence) in TPGS-treated HCC cells, scale bar = 100 μm. (**B**) Quantitative analysis of ROS production in TPGS-treated HCC cells. The 44 μM TPGS group had the highest ROS positive cell rate, 6.0%. (**C**) ΔΨ levels were analysed in HCC cells to evaluate energy production. (**D**) Effects of TPGS treatments on NOS activity. (**E**) TPGS induced an increase of cleaved PARP protein levels and LC3-II protein accumulation in TPGS-treated HCC cells. (**F** and **G**) Quantitative analysis of western blot results from (**E**). All protein levels were normalized with the housekeeping genes GAPDH and β-actin.

In fact, most anticancer agents kill cancer cells by augmenting ROS stress [21]. To determine the intrinsic mechanism of TPGS-induced apoptosis, we first determined the ROS levels in TPGS-treated HCC cells (Figure 5A and Supplementary Figure 2C, 2D). As we inferred, TPGS dose-dependently induced the production of ROS in HepG2 cells from 0% to approximately 6% (Figure 5B). It is known that endogenous ROS are produced through multiple mechanisms, and a major source is the mitochondrion [22]. An impaired respiratory chain may induce incomplete oxidation-reduction reactions to produce the superoxide radical (·O_2_^−^), which is the precursor of most other ROS. Also, the mitochondrial membrane potential (ΔΨm) is critical for maintaining the physiological function of the respiratory chain [23]. As shown in Figure 5C and Supplementary Figure 2E, the ΔΨm of HepG2 cells decreased almost 92% in response to TPGS treatment, which suggests that the occurrence of apoptosis induced by TPGS is due to a bioenergetic imbalance. Alternatively, endogenous NO that is mainly produced by NOS has also been reported to induce apoptosis in different cell systems such as epithelial and endothelial cells, as well as in cancer cells [24]. However, as shown in Figure 5D, the NOS activity in TPGS-treated cells not only did not rise, but decreased to some extent (approximately 9%). This result indicates that TGPS treatments significantly impaired the function of mitochondria, which play a central role in maintaining HCC cell homeostasis, and that NO- related pathways were not the main cause of the anti-HCC effects of TPGS.

### TPGS treatments effectively enhance PARP and LC3-II expression in HCC cells

As described above, TPGS treatments induced the generation of ROS in HCC cells as well as massive cell apoptosis. To investigate the molecular pathway behind this apoptosis, we next examined the expression of the cell apoptosis-related protein Poly (ADP-ribose) polymerase (PARP). As shown in Figure 5E, 5F, cleaved PARP accumulated in TPGS-treated HCC cells (an approximate 44% increase), which could promote apoptosis by preventing DNA repair-induced survival and by blocking energy depletion-induced necrosis [25, 26]. In addition to apoptosis, we examined autophagy, another important intracellular process related to cancer in HCC cells. As a result, the levels of the autophagy marker LC3-II rise in a dose-dependent manner (Figure 5E, 5F), indicating that TPGS can enhance the formation of autophagosomes. Furthermore, Erk/pErk and β-catenin western blots indicated no obvious changes, indicating that MEK-ERK and Wnt-β-catenin signalling pathways were not involved in the TPGS induced anti-HCC effects (Figure 5E and 5G).

### TPGS treatment inhibited liver tumor growth *in vivo*

**Figure 6:**
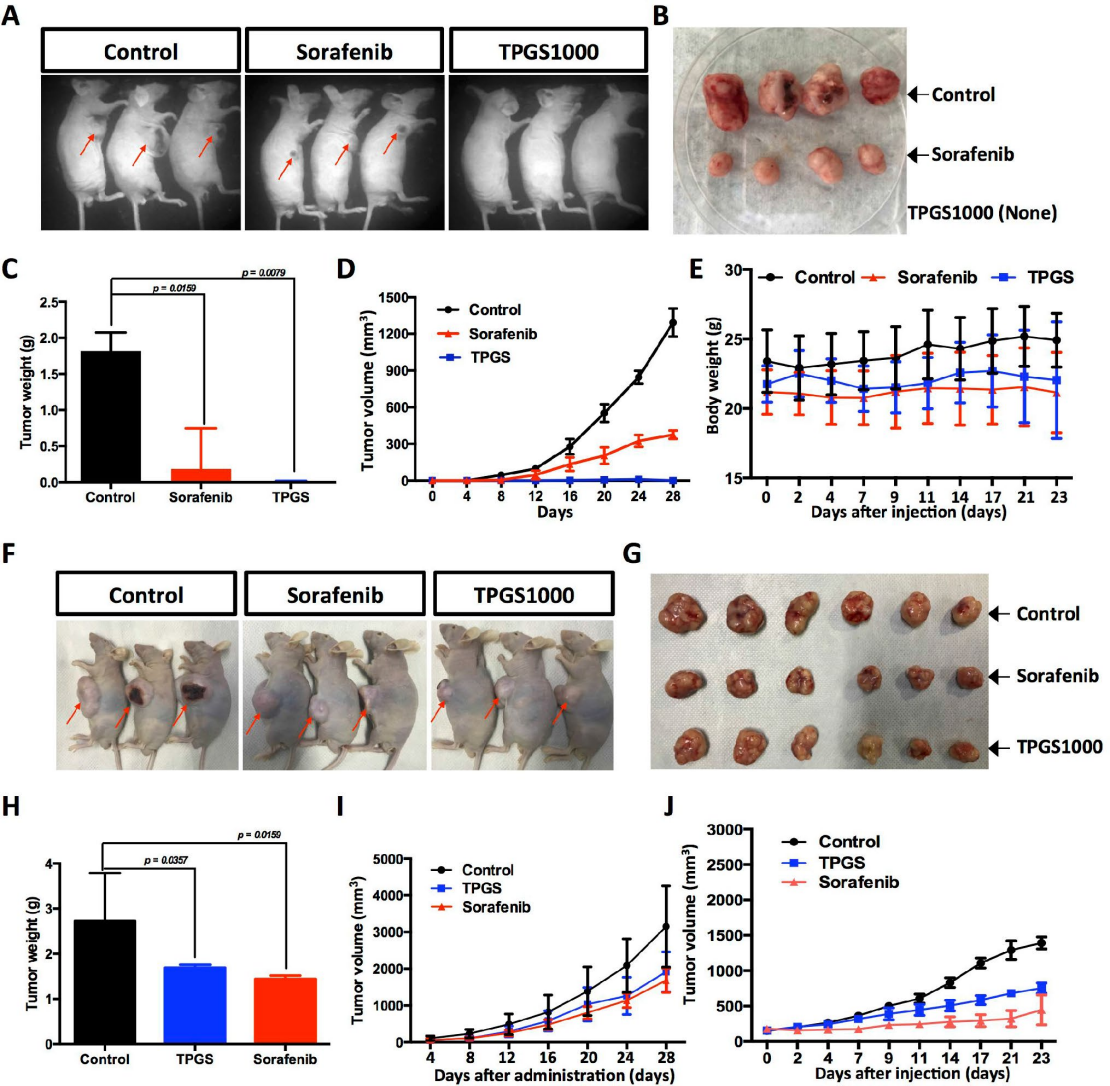
**Effects of TPGS treatments and intravenous or oral administration on HCC cell-derived subcutaneous xenograft tumors in nude mice.** (**A**) Tumor-bearing mice were divided into three groups: control, Sorafenib- and TPGS-treated groups. (**B**) Tumor masses from the control group (implantation with 1×107 untreated HCC cells) and from the Sorafenib group (implanted with 1×107 treated HCC cells). (**C**) Tumor weights in mice 32 days after injection. (**D**) Quantitative analyses of tumor progression of (**A**). The tumor size was determined by measuring the tumor volume every 4 days from day 4 to day 28 after injection. (**E**) Body weights for TPGS-injected animals did not change significantly compared to vehicle-treated controls. (**F**) Tumor-bearing mice were randomized to receive treatment with 30 mg/kg of Sorafenib or 300 mg/kg of TPGS or an equal volume of normal saline by oral gavage. (**G**) Tumor masses from three groups of (**F**). (**H**) Tumor weights in mice 32 days after drug administrations. Both the Sorafenib- and TPGS-treated groups demonstrated a significant decrease of tumor weights. (**I**) Quantitative analyses of tumor progression of (**F**). (**J**) Tumor growth curves. Nude mice were administered treatments 2 times via intravenous injection in the tail over an interval of 7 days. The tumor volume was monitored at predetermined time points.

To investigate the anti-HCC effect of TPGS *in vivo*, we established a subcutaneous xenograft tumor model of HCC cells (Figure 6A). Vehicle control, Sorafenib-treated and TPGS-treated HCC cells were injected into nude mice. Tumor volumes were measured at different time points of tumor growth in various groups. After 32 days, tumors in each mouse were removed and weighed (Figure 6B). Compared with the control group, both Sorafenib and TPGS treatments significantly decreased the solid tumor mass (Figure 6B, 6C), indicating that TPGS treatment could significantly suppresses liver tumor formation *in vivo*. From the timeline, mice injected with untreated HepG2 cells did not exhibit an obvious increase in the mean tumor size compared with the Sorafenib- and TPGS-treated groups in the first 8 days. However, after 12 days, three groups yielded separate tumor growth curves. TPGS treatment completely inhibited tumor formation during the 28 days, followed by Sorafenib (Figure 6D).

Considering the absorption and metabolism of TPGS *in vivo*, we conducted oral administration of TPGS and Sorafenib when the subcutaneous tumors reached a volume of ~100 mm^3^. Like Sorafenib, TPGS treatment resulted in a significant reduction in the growth of xenografts compared with vehicle control (Figure 6F–6I). To evaluate the *in vivo* safety and anti-tumor efficacy of TPGS by intravenous administration, we further plotted the body weight and tumor growth curves in intravenous drug testing. As shown in Figure 6E and 6J, TPGS began to exert tumor suppression after the second injection (Figure 6J), and did not cause significant changes in the body weights (Figure 6E).

## DISCUSSION

As an FDA approved pharmaceutic adjuvant, TPGS is chemically stable with a hydrophilic head (polyethylene glycol chain) and a lipophilic tail (phytyl chain of d-alpha-tocopherol), and has been widely applied as a vehicle for drug delivery to enhance drug solubility and increase the oral bioavailability of anticancer drugs (Figure 1B) [13, 27–29]. TPGS alone has been reported to induce cell death in lung, breast, prostate and blood cancers [11, 14, 30, 31]. However, the extent of its anticancer effect in liver cancer cells remains unclear. In this study, our results indicate that TPGS treatment dose-dependently inhibits HCC cell proliferation by arresting the cell cycle in the G0/G1 phase (Figure 3A and 3C) and by inducing apoptotic cell death (Figure 3B, 3D, 3E, and 4B and 4D). Additionally, due to its amphiphilic structure and enhanced permeability and retention effects, TPGS significantly suppressed the migration and invasion of HCC cells accompanied by its cytotoxicity (Figure 2 and Supplementary Figure 1-2), which suggests that TPGS could be used as a promising therapeutic agent to prevent metastasis in liver cancer.

Among all of the natural health supplements, vitamin E is probably the most intensively studied cancer preventive agent because of its renowned antioxidant property [32, 33]. However, TPGS, a succinyl derivative of vitamin E that differs in general from vitamin E itself, does not act as an antioxidant [34]. The anticancer activity of TPGS is mediated by its unique apoptosis-inducing properties [11], which appear to be mediated through diverse mechanisms involving the generation of ROS [14, 30]. Our results confirmed this speculation in HCC cells. That is, TPGS can induce a decrease in the mitochondrial membrane potential (ΔΨm) in HCC cells (Figure 5C), which can further trigger ROS production by leaking electrons from the mitochondrial respiratory chain [35] and the reaction of these electrons with O_2_ (Figure 5A and 5B). Following mitochondrial dysfunction, ROS-associated damages in DNA, proteins and lipids [36] result in the accumulation of cleaved PARP (Figure 5E) and progressive HCC cell apoptosis.

From the perspective of downstream signalling molecules, our findings demonstrate that TPGS induced excessive generation of ROS, which may further downregulate the transcription factor FoxM1, a critical sensor and regulator of oxidative stress during oncogenesis (Figure 4E, 4F) [37]. As FoxM1 is implicated in the negative regulation of cell cycle inhibitors p21 [38], a reduced expression and phosphorylation of FoxM1 with a concomitant increase in p21 leads to a blockage of the G1-S phase transition, which produces a decrease in S phase cells (Figure 3A and 3C) as well as cell cycle arrest in the G0/G1 phase. Instead, overexpression of FoxM1 in TPGS treated HCC cells again brings p21 back to the normal level (Supplementary Figure 3C, 3D). In addition, consistent with previous reports in pancreatic cancer [39], we also observed inhibition of migration and invasion by FoxM1 downregulation that was induced by TPGS treatment in HCC cells.

Furthermore, in our experiments, TPGS elevated the protein levels of LC3-II in HepG2 cells, a reliable marker for assessing autophagic flux (Figure 5E). Although the interplay of autophagy and cancer remains ambiguous and controversial, it is clear that autophagy is deeply integrated into metabolism, stress responses and cell death pathways [40]. Recent investigations have suggested that LC3 can facilitate prognosis of HCC [41], which confirmed our result that an elevated LC3 level is beneficial in the treatment of HCC. Another interesting finding is that the NOS activity in HCC cells was down-regulated by TPGS (Figure 5D). This likely is due to the loss of mitochondrial NOS (mtNOS) with mitochondrial dysfunction. Previous studies indicated that NO increases the accumulation of p53, leading to growth arrest and apoptosis [42]. Our result suggests that the effects of TPGS on liver cancer do not occur by the activation of NO signalling.

Studies in animal models of HCC xenografts have found that TPGS can be safely used as an intravenous administration (Figure 6E), which is in accord with earlier reports that TPGS exhibits selective cytotoxicity for cancer cells compared with their normal counterparts [14]. We speculate that one probable reason is that tumor cells are generally more sensitive to oxidative stress compared with normal cells [43]. In addition, we noticed that the effect of TPGS on the HCC model by oral or intravenous administration (Figure 6I and 6J) was not as significant as it was in *in vitro* direct treatment. We anticipate improving the therapeutic outcome by increasing the number of TPGS administrations from two to three or more. Recently, TPGS was shown to inhibit the function of P-glycoprotein, a protein that causes MDR by acting as a drug efflux pump [44], which suggests that TPGS may be used as an anti-HCC enhancer. Taking all of this into consideration, a better understanding of the detailed mechanisms involved in the anti-HCC efficacy of TPGS requires further investigation.

In summary, our study demonstrated that TPGS inhibits HCC cell viability and mobility by impairing mitochondrial function by activating FoxM1, p21, PARP and LC3 signalling. These findings suggest that TPGS could be used as a promising agent for the treatment of liver cancer.

## MATERIALS AND METHODS

### Chemicals and antibodies

TPGS1000 (Figure 1B) was purchased from Sigma (Sigma-Aldrich Corp., St. Louis, MO USA) and was dissolved in Dulbecco's phosphate-buffered saline (DPBS) at a stock concentration of 20 mM and stored at −20°C. Fresh dilutions in medium were prepared for each experiment.

Anti-FoxM1 rabbit antibodies were obtained from Abcam (Abcam, USA); Anti-LC3 and anti-p21 mouse antibodies were obtained from HuaBio (HuaBio, Hangzhou, China); anti-GAPDH mouse mAb was obtained from Sangon (Sangon Biotech, Shanghai, China); anti-cleaved-PARP, anti-Erk1/2, anti-pErk1/2, anti-CNR1, anti-β-actin and anti-β-catenin antibodies were purchased from CST (Cell Signalling Technology Inc., Danvers, MAUSA). Unless specifically stated, all other reagents were purchased commercially.

### Cell culture

HepG2, Hep3B, Huh7 and Bel7402 HCC cells were obtained from the American Type Culture Collection (ATCC) and the Shanghai Cell Bank of the Chinese Academy of Sciences (CAS) and were maintained in a humidified incubator at 37 °C in a 5% CO2 atmosphere in Dulbecco’s Modified Eagle’s Medium (DMEM, Gibco, Grand Island, NY, USA) supplemented with 10% foetal bovine serum (FBS, Gibco) and antibiotics (Gibco). Cells were passaged three times a week after becoming confluent. For confocal microscopy, the cells were seeded onto 12 mm×12 mm glass coverslips (Thermo Fisher Scientific, Waltham, MA, USA) at various densities.

### Cell proliferation and CCK8 assay

HCC cells were treated with various concentrations (0–66 μM) of TPGS and were seeded into 96-well plates at 5000 cells per well. After 48 h, 10 μL of CCK8 were added to each well and the cells were incubated at 37°C for an additional 1 h [45]. The optical absorption values were then measured at 450 nm and the data are presented as the means ± standard deviation (SD), which were derived from triplicate samples from at least three independent experiments. In parallel, cell growth curves were also plotted based on cell counting [46] under different concentrations of TPGS (0, 11, 22, 44 μM).

### Cell migration assay

Cell migration was determined using a wound healing and a Transwell assay [47]. For the wound-healing assay, HCC cells (1 × 10^6^/mL/well) at passage 3–4 were serum-starved for 24 h and then seeded into six-well plates and allowed to adhere for 12 h. Confluent monolayer cells were scratched with a sterile 200 μL pipette tip. The cells were washed with DPBS to clear debris and suspended cells. Fresh serum-free medium containing different concentrations of TPGS was added, and the wounds were recorded under a phase contrast microscope at 0 and 24 h. Migration distance was calculated from the change in wound size during a 24 h period using ImageJ software. For Transwell assays, HCC cells (5×10^4^) were suspended in serum-free medium containing different concentrations of TPGS. Transwell insert chambers (Corning Life Sciences, Corning, NY, USA) with 8 μm pore filters were used. Cells were seeded on the top chambers of the wells in 200 μL of medium, and 600 μL of 10% FBS medium were added to the lower chambers to induce cell migration. After 24 h of incubation, the cells on the filter surface were fixed in 4% PFA for 1 h at room temperature and stained with a Crystal violet solution for 20 min at room temperature. Finally, the number of violet cells were counted.

### Transwell invasion assays

Cell invasion ability was measured using a Transwell invasion assay. Briefly, HCC cells (5×10^4^) were suspended in serum-free medium containing different concentrations of TPGS. Transwell insert chambers (Corning Life Sciences, Corning, NY, USA) with 8 μm pore filters were coated with 0.5 mg/mL Matrigel (BD Sciences, Bedford, MA, USA). Cells were seeded on the top chambers of the wells in 200 μL of medium, and 600 μL of 10% FBS medium were added to the lower chambers to induce cell invasion. After 24 h of incubation, the cells on the filter surface were fixed in 4% PFA for 1 h at room temperature and stained with a Crystal violet solution for 20 min at room temperature. Finally, the number of violet cells were counted.

### Annexin V-7-AAD apoptosis assay

Cell apoptosis was assessed by Annexin V-FITC and Propidium (PI) staining (BD Sciences). The treated cells were collected and washed three times with DPBS, and then incubated in 200 μL of staining solution containing Annexin V-FITC antibody and PI for 15 min in the dark at room temperature. The cells were analysed immediately with a Cantos flow cytometer (Becton Dickinson, Mountain View, CA, USA) and Cell Quest Pro software. For each measurement, at least 2×10^4^ cells were counted and the cell apoptosis rate was determined in three independent experiments.

### Flow cytometric analysis of the cell cycle

Cell cycle analysis was performed using propidium iodide (PI) staining for DNA quantitation [48]. Cells were harvested, washed and centrifuged at 1000 rpm for 5 min, and subsequently fixed in 70% ethanol at 4°C for more than 1 h, followed by washing with DPBS. Cells were then resuspended in 400 μL of DPBS containing 0.05% Triton X-100, 0.1 mg/mL DNase-free RNase A, and 25 μg/mL PI and incubated for 30 min at 37 °C in the dark. For each measurement, at least 2×10^4^ cells were analysed using a Cantos flow cytometer. The cell cycle data were processed using Cell Quest Pro software.

### EdU and TUNEL staining

EdU (5-ethynyl-2’-deoxyuridine) cell proliferation assays and Terminal Transferase and Biotin-16-dUTP (TUNEL Fluorescent assay) were performed with a commercially available EdU staining kit (Ribobio, Guangzhou, China) and a TUNEL staining kit (Roche Applied Sciences) by following the manufacturer's instructions, including the use of positive and negative controls. HCC cells containing different concentrations of TPGS were subjected to the assays and counter-stained with DAPI.

### Western blot analysis

For western blotting, proteins were extracted with RIPA buffer (50 mM Tris–HCl, pH 7.4, 1% Triton X-100, 0.25% sodium deoxycholate, 150 mM NaCl, 1 mM EDTA, 0.1% SDS and a protease inhibitor cocktail) and separated by SDS-PAGE. The resolved proteins were transferred to PVDF membranes (Millipore). Nonspecific reactivity was blocked by incubating the membrane in 10 mM Tris–HCl (pH 7.5), 150 mM NaCl, 2% Tween 20 and 4% bovine serum albumin (BSA) for 1 h at 37°C. Diluted primary antibody was then added, followed by the appropriate secondary antibody. Proteins were detected using the enhanced chemiluminescence (ECL) system (Thermo Fisher Scientific) [49].

### Plasmid transfection

A pCMV-FoxM1-Hygro plasmid (Sino Biological, Beijing, China) was used to construct the FoxM1-expressing vector. The HCC cells were transfected with Lipofectamine 3000 (Thermo Fisher Scientific) following the manufacturer's instructions.

### Reactive oxygen species (ROS) and Nitric Oxide Synthase (NOS) activity assays

Cellular ROS and NOS activities were determined using an ROS detection assay kit (Solarbio, China) and an NOS assay kit (Beyotime, Haimen, China) by following the instruction manual. Briefly, oxidative stress (total ROS) in living cells can be determined using the green fluorescent dye DCF and Fluorescence Microscopy (Nikon TRE, Japan). In the NOS assay, nitric oxide that is generated by NOS undergoes a series of reactions to generate a coloured product that has a strong absorbance at 540 nm, which can be measured by a microplate reader (Molecular Devices, USA).

### Mitochondrial Membrane Potential Assay

JC-10 (Yesen, Shanghai, China) is capable of selectively entering mitochondria, and reversibly changes its colour from green to orange as membrane potentials (ΔΨm) increase. The green emission can be analysed in the fluorescence channel 1 (FL1) and the orange emission in channel 2 (FL2) with a flow cytometer. The ΔΨm was calculated from the JC-10 aggregate ratio (orange/green) and analysed by GraphPad Prism 5.01 (GraphPad Software Inc, La Jolla, CA, USA).

### Tumor growth assay *in vivo*

Four or five week old immune-deficient nude mice (BALB/c-nu) were purchased from the Shanghai SLAC Laboratory Animal Company. The mice were maintained in the facility for laboratory animals at the Hangzhou Normal University. The protocol for the experiment was approved, and animals were handled according to the ethical standards of the Institutional Animal Care and Use Committee of the Hangzhou Normal University. The mice were assigned randomly to 1 of 3 groups (Supplementary Table 1). For direct drug effect study, Control (DMSO), Sorafenib (5 μM) and TPGS (22 μM)-treated HepG2 cells were harvested and counted. 1×10^7^ cells were injected subcutaneously into the right flank of the nude mice, which led to palpable nodules on day 16. For oral administration study, mice carrying 100 mm^3^ subcutaneous tumors were randomized to receive 4 batches of treatment with 30 mg/kg of Sorafenib or 300 mg/kg of TPGS or an equal volume of normal saline by oral gavage. The tumor volume was measured with callipers every 4 days throughout the observation period of 4 weeks and calculated using the formula: Volume = length × width^2^ × 0.5 [50]. All of the mice were sacrificed on day 32 and the tumor weights were measured.

### Evaluation of the safety and efficacy of intravenous TPGS

The *in vivo* safety and anti-tumor efficacy of TPGS were evaluated in Bel7402 cell-bearing BALB/c nude mice. Cells (10^7^) were injected subcutaneously into the right flanks of nude mice to establish Bel7402 xenografts. The treatment was initiated when the tumor volume reached approximately 100 mm^3^. In the tumor model, mice were randomly divided into two groups (n=8): the DPBS group (control group, n=8) and the TPGS group with a dose of 100 mg/kg through tail-vein injections. The mice were treated every 7 days for a total of two injections. Tumor volumes and mouse body weights were monitored at predetermined time points.

### Statistics

In addition to the special notes, the data were analysed using SPSS12.0 and expressed as the median plus range. Statistical comparisons between two groups were made using a nonparametric Mann-Whitney U test, and probability values (*p*) < 0.05 were considered significant.

## Supplementary Material

Supplementary Figures

Supplementary Tables
