# Supplementary material for: TPGS-1000 exhibits potent anticancer activity for hepatocellular carcinoma in vitro and in vivo
Source: Aging (Albany NY). 2020 Jan 27;12(2):1624–42. doi: 10.18632/aging.102704 (PMC7053644; doi:10.18632/aging.102704)
Supplement: Supplementary Figures [file aging-12-102704-s002..pdf]

SUPPLEMENTARY FIGURES

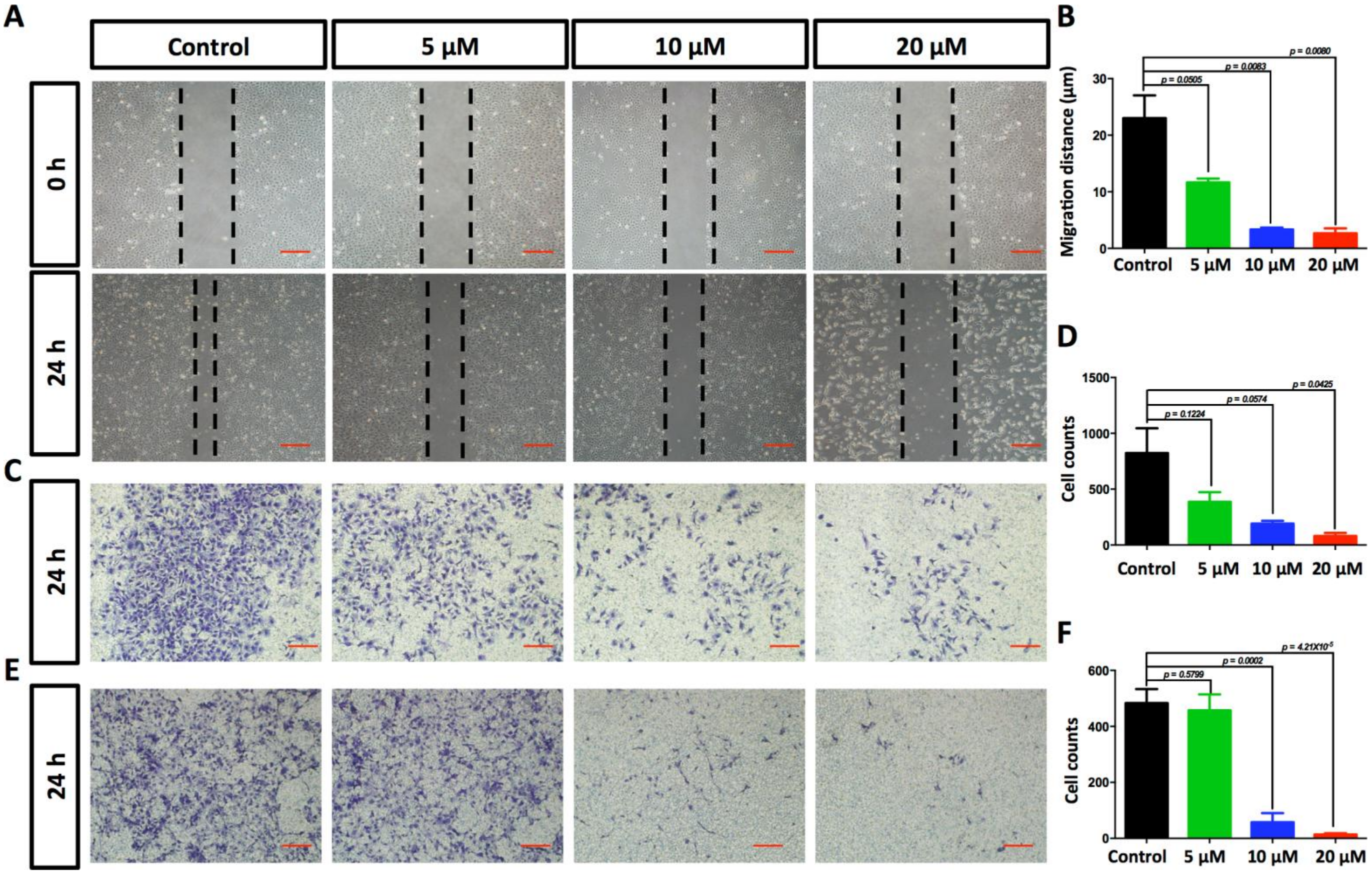

Supplementary Figure 1. TPGS dose dependently restrained Hep3B cell migration and invasion. (**A**) Effects of TPGS treatments on Hep3B cell migration, scale bar = 50 μm (**B**) The migration distance of Hep3B cells was quantified by ImageJ software, and the 20 μM TPGS group had the shortest migration distance (3 μm). (**C**) The inhibition of Hep3B cell migration by TPGS was confirmed by Transwell assays, scale bar = 100 μm. (**D**) The migrated cells were counted after Crystal violet staining with the 20 μM TPGS group having the lowest number of migrated cells (approximately 80). (**E**) TPGS diminished cell invasion of Hep3B cells (Transwell assay using an 8 μm pore filter coated with 0.5 mg/mL Matrigel), scale bar = 100 μm. (**F**) The mean cell counts of invading cells with the 20 μM TPGS group having the lowest number of invasion cells (approximately 13).

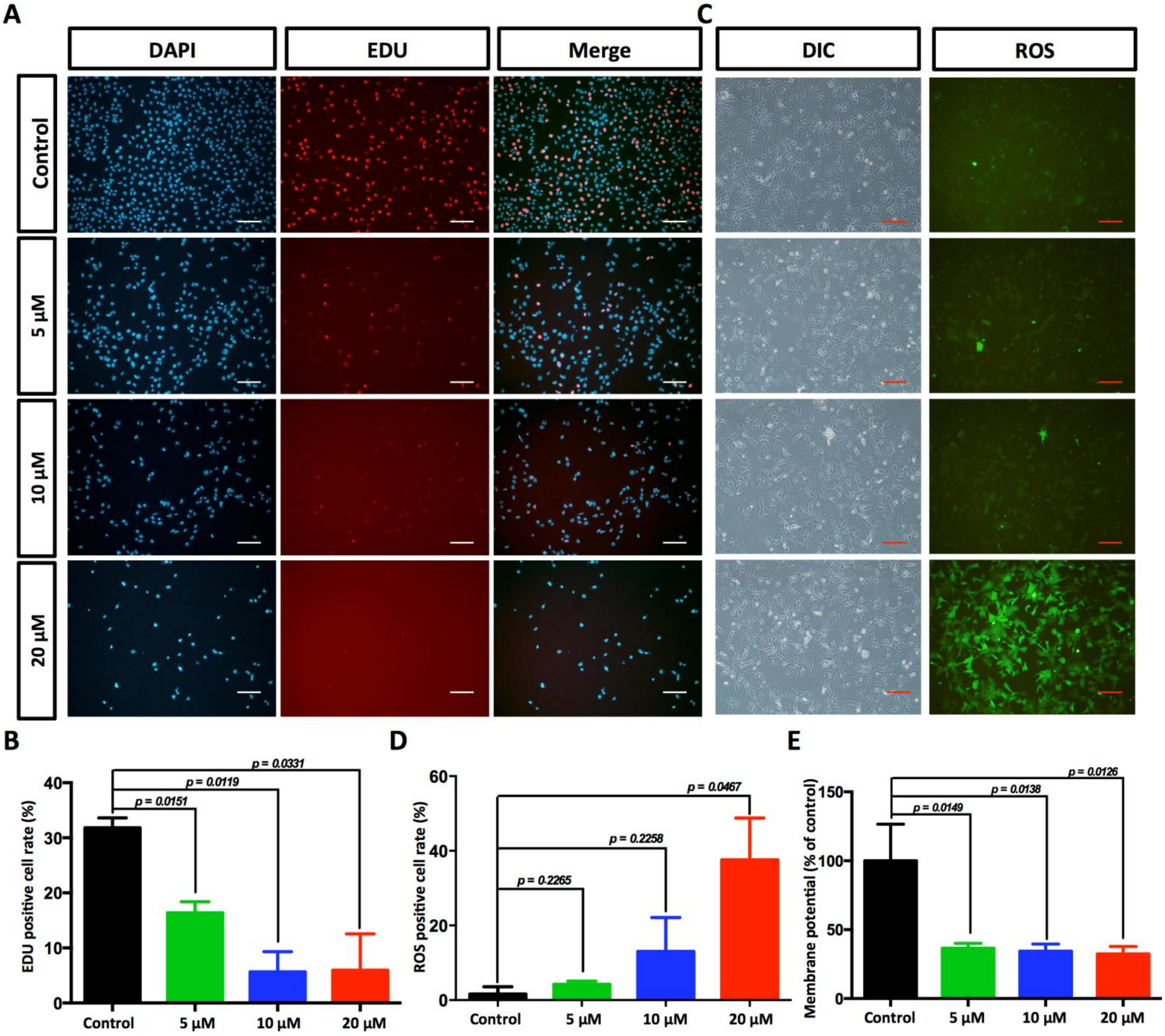

Supplementary Figure 2. Suppression of DNA synthesis and induced the production of ROS in TPGS-treated Hep3B cells. (**A**) Detection by fluorescence microscopy of EdU (red) incorporated into the DNA of cultured Hep3B cells, scale bar = 40 μm. The nuclei were counter-stained with DAPI (blue). (**B**) The rates of EdU positive cells that passed through the S phase were calculated with ImageJ, and the 20 μM TPGS group had the lowest EdU positive cell rate (6%). (**C**) ROS imaging (green fluorescence) in TPGS-treated Hep3B cells, scale bar = 100 μm. (**D**) Quantitative analysis of ROS production in TPGS-treated Hep3B cells. (**E**) $\Delta\Psi$ levels were analysed in Hep3B cells to evaluate energy production.

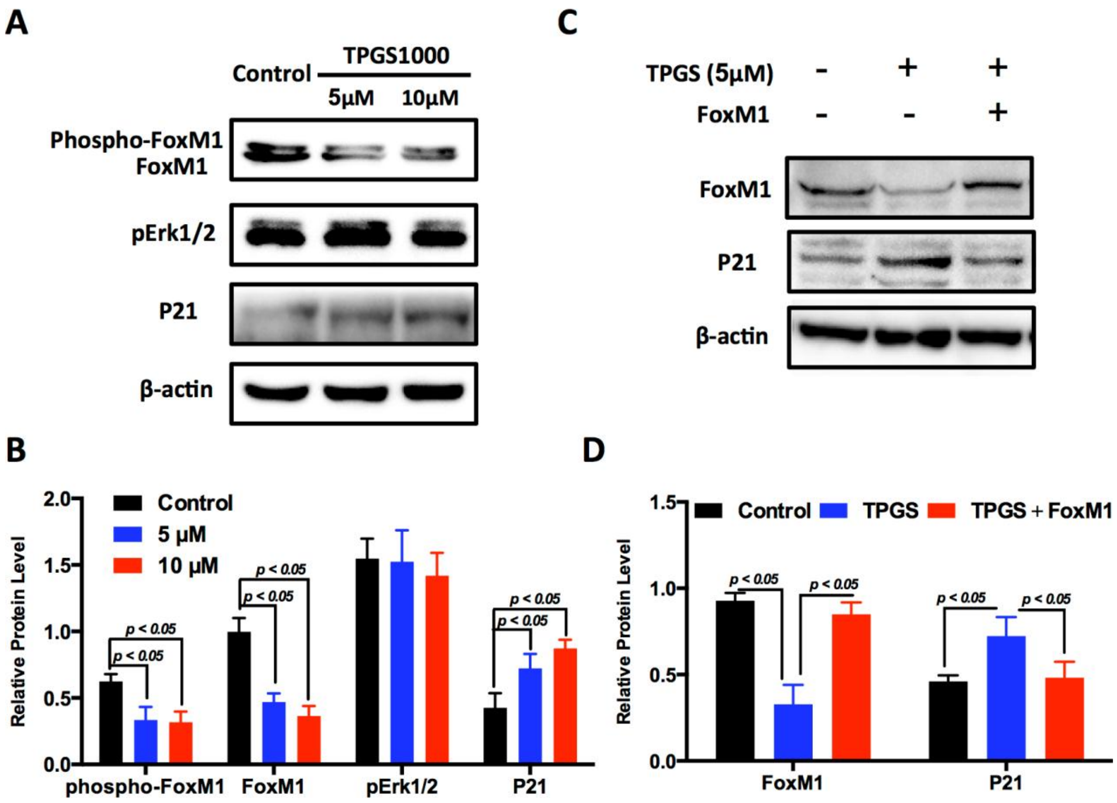

Supplementary Figure 3. FoxM1-p21 signalling mediates the anti-HCC effects of TPGS. (**A**) A decrease of FoxM1 and phosphorylated FoxM1, and an increase of p21 protein levels in TPGS-treated Hep3B cells. (**B**) Quantitative analysis of western blot results from (**A**). (**C**) Overexpression of FoxM1 in TPGS treated HepG2 cells again brings p21 back to the normal level. (**D**) Quantitative analysis of western blot results from (**C**). All protein levels were normalized with the housekeeping gene β-actin.
