# Supplementary material for: TPGS-1000 exhibits potent anticancer activity for hepatocellular carcinoma in vitro and in vivo
Source: Aging (Albany NY). 2020 Jan 27;12(2):1624–42. doi: 10.18632/aging.102704 (PMC7053644; doi:10.18632/aging.102704)
Supplement: Supplementary Tables [file aging-12-102704-s001..pdf]

SUPPLEMENTARY TABLES

Supplementary Table 1. Animals.

Administration methods	Control	TPGS	Sorafenib
Tumor cell treatment	n=4	n=4	n=4
Tail vein injection	n=6	n=6	n=6
Oral administration	n=6	n=6	n=6

Supplementary Table 2. List of predicted targets for TPGS in HCC cells.

Target	Uniprot ID	Gene Code	Probability	#sim. cmpds (3D/2D)	Target Class
Prostaglandin G/H synthase 1	P23219	PTGS1	0.5	2/7	Enzyme
Prostaglandin G/H synthase 2	P35354	PTGS2	0.5	2/7	Enzyme
Cannabinoid receptor 1	P21554	CNR1	0.5	28/296	Membrane receptor
Cannabinoid receptor 2	P34972	CNR2	0.5	18/249	Membrane receptor
Potassium voltage-gated channel subfamily H member 2	Q12809	KCNH2	0.37	9/15	Ion channel
Potassium voltage-gated channel subfamily H member 6	Q9H252	KCNH6	0.37	9/15	Ion channel
Potassium voltage-gated channel subfamily H member 7	Q9NS40	KCNH7	0.37	9/15	Ion channel
Estrogen receptor	P03372	ESR1	0.35	3/73	Transcription Factor
Estrogen receptor beta	Q92731	ESR2	0.35	3/73	Transcription Factor
Squalene monooxygenase	Q14534	SQLE	0.27	2/4	Enzyme
Histone deacetylase 1	Q13547	HDAC1	0.22	3/5	Enzyme
Histone deacetylase 3	O15379	HDAC3	0.22	3/5	Enzyme
Histone deacetylase 2	Q92769	HDAC2	0.22	3/5	Enzyme
Histamine H3 receptor	Q9Y5N1	HRH3	0.22	2/32	Membrane receptor
D (2) dopamine receptor	P14416	DRD2	0.2	18/129	Membrane receptor
